# Diarrhœa of Dentition

**Published:** 1894-06

**Authors:** 


### Diarrhœa of Dentition. (Treatment.)

[Meigs—*Med. Rec.*]

—MIXTURE.—

| | |
|---|---|
| Dilute sulphuric acid | 12 drops, |
| Morphine sulphate | ¼ grain (16 mg.), |
| Cognac | of each, ½ fl. oz. (15 c.c.), |
| Syrup ginger | |
| Distilled water | enough to make 3 " (89 " ), |

One fluid dram. (3.75 c.c.) every three hours.

Chloroform is recommended as being one of the best remedies for the controlling of external hemorrhage, whether arterial, venous or capillary. It may be applied with lint or cotton.

